# Assessment of a Reliable Fractional Anisotropy Cutoff in Tractography of the Corticospinal Tract for Neurosurgical Patients

**DOI:** 10.3390/brainsci11050650

**Published:** 2021-05-16

**Authors:** Tim Wende, Johannes Kasper, Florian Wilhelmy, Eric Dietel, Gordian Hamerla, Cordula Scherlach, Jürgen Meixensberger, Michael Karl Fehrenbach

**Affiliations:** 1 Department of Neurosurgery, University Hospital Leipzig, Liebigstr. 20, 04103 Leipzig, Germany; johannes.kasper@medizin.uni-leipzig.de (J.K.); florian.wilhelmy@medizin.uni-leipzig.de (F.W.); eric.dietel@medizin.uni-leipzig.de (E.D.); juergen.meixensberger@medizin.uni-leipzig.de (J.M.); michael.fehrenbach@medizin.uni-leipzig.de (M.K.F.); 2 Department of Neuroradiology, University Hospital Leipzig, Liebigstr. 20, 04103 Leipzig, Germany; gordian.hamerla@medizin.uni-leipzig.de (G.H.); cordula.scherlach@medizin.uni-leipzig.de (C.S.)

**Keywords:** neurosurgery, tractography, glioma, fractional anisotropy

## Abstract

Background: Tractography has become a standard technique for planning neurosurgical operations in the past decades. This technique relies on diffusion magnetic resonance imaging. The cutoff value for the fractional anisotropy (FA) has an important role in avoiding false-positive and false-negative results. However, there is a wide variation in FA cutoff values. Methods: We analyzed a prospective cohort of 14 patients (six males and eight females, 50.1 ± 4.0 years old) with intracerebral tumors that were mostly gliomas. Magnetic resonance imaging (MRI) was obtained within 7 days before and within 7 days after surgery with T1 and diffusion tensor image (DTI) sequences. We, then, reconstructed the corticospinal tract (CST) in all patients and extracted the FA values within the resulting volume. Results: The mean FA in all CSTs was 0.4406 ± 0.0003 with the fifth percentile at 0.1454. FA values in right-hemispheric CSTs were lower (*p* < 0.0001). Postoperatively, the FA values were more condensed around their mean (*p* < 0.0001). The analysis of infiltrated or compressed CSTs revealed a lower fifth percentile (0.1407 ± 0.0109 versus 0.1763 ± 0.0040, *p* = 0.0036). Conclusion: An FA cutoff value of 0.15 appears to be reasonable for neurosurgical patients and may shorten the tractography workflow. However, infiltrated fiber bundles must trigger vigilance and may require lower cutoffs.

## 1. Introduction

Tractography of the corticospinal tract has become a routine technique for planning neurosurgical procedures in the eloquent areas of the brain. However, tractography still suffers from uncertain accuracy and reproducibility [1,2]. There is increasing evidence that, if correctly applied, tractography can reliably predict the position of fiber bundles to a degree of some millimeters [3]. In particular, knowledge of the beginning and the end of the bundle is important, so that the combination with transcranial magnetic stimulation (TMS) or functional magnetic resonance imaging (fMRI) enriches the technique with the information of primary functions and more accurate localization [4,5,6,7]. This has also been verified by intraoperative direct electrical stimulation [8,9].

After defining the regions of interest (ROIs), a tracking algorithm must be chosen and adequately configured. Different tracking algorithms can deliver visually different results that must be interpreted with caution and with knowledge of the technical process. Today, probabilistic algorithms are predominant [3,10]. While certain parameters, such as the number of seeds, depend on the computing capacity, others, like the minimal and maximal streamline length, are anatomically determined. However, a parameter of outstanding importance is the cutoff value for the fractional anisotropy (FA) in a streamline.

The FA is a measure for the directedness of diffusion, with values between 0 and 1, and is low in fluids and high in fiber bundles [11]. In iPlan Cranial 3.0 (Brain Lab AG, Munich, Germany), a prevalent software tool for neurosurgical planning, the standard cutoff is set to 0.3 [12,13,14]. In recent studies, this cutoff has been individually determined by a structured process of trial and error, with the lowest value at 0.1 [6,15]. 

This process delivers acceptable results and also considers the individual anatomy of patients with intracerebral lesions and perifocal edema, as well as different diffusion MRI protocols. However, there is no sufficient data yet to deem it the most sufficient tractography workflow. In practice, neuroradiologists and neurosurgeons require a verified starting point for the tractography process that they already use in their daily work. We, therefore, analyzed a cohort of 14 patients undergoing craniotomy for intracerebral lesions and searched for a statistically meaningful FA cutoff in the corticospinal tract after applying our tractography workflow.

## 2. Materials and Methods

### 2.1. Patients

Patients were included after obtaining written informed consent. The study was approved by the local ethics committee (No. 322/19-ek) and registered at ClinicalTrials.gov (NCT04302857). The inclusion criteria were the intracerebral location of the tumor and age between 18 and 75 years. The exclusion criteria were a cerebral ischemia or spontaneous intracerebral hemorrhage, pregnancy, and encephalitis.

### 2.2. Image Acquisition

An MRI was obtained within seven days before as well as within seven days after surgery. All images were acquired with the same 3T scanner (Ingenia, Philips Nederland B.V., Eindhoven, Netherlands) using a single-shot echo planar imaging diffusion tensor imaging (DTI) sequence (TR/TE = 7010/102 ms; FOV = 222 × 222 mm^2^; matrix 112 × 112; 50 slices without gap; slice thickness 2.7 mm; 32 non-colinear directions; and b-value = 1000 s/mm^2^), a high-resolution T1 weighted turbo field echo (TFE) sequence (TR/TE = 8.1/3.7 ms; FOV = 222 × 222 mm^2^; matrix = 512 × 512; 170 slices without gap; and slice thickness 1 mm), and a dedicated head coil.

### 2.3. Image Processing

The data were processed on an Apple computer (iMacPro1,1, Intel Xeon W 10C 3.0 GHz, 64 GB RAM, 1 TB SSD, and AMD Radeon Pro Vega 64X). Dicom images were converted using *dcm2niix* [16] and *mrconvert* [17]. After calculating diffusion tensor images from diffusion-weighted raw data with *dwi2tensor*, we calculated FA images with *tensor2metric* and created fiber orientation distribution (FOD) images by constrained spherical deconvolution (CSD) with *dwi2fod*. We performed the last step with a mask image, which we created with *dwi2mask* [17,18].

### 2.4. Tractography

Streamline tractography was performed with *tckgen* in MRtrix3 with a probabilistic algorithm based on second order integration over fiber orientation distribution (iFOD2) [19]. Regions of interest (ROIs) were located in the T1 image axial slices. The seed ROI was placed anterolaterally in the mesencephalon at the height of the cavernous sinus with a radius of 5 mm. The target ROI was placed between the anterior two-thirds of the internal capsule′s posterior limb with a radius of 10 mm. The number of seeds was 10^6^ in each hemisphere. The minimum streamline length was set to 30 mm, and the maximum streamline length was set to 250 mm. 

Our arbitrary FA cutoff was 0.2. The tractogram was examined by an experienced neurosurgeon and an experienced neuroradiologist. We excluded streamlines crossing to the contralateral hemisphere. We further excluded collaterals to other fiber bundles, which could be clearly identified by their anatomical configuration, such as the inferior fronto-occipital fascicle, cerebellar collaterals, or the arcuate fascicle. Only streamlines that were clearly not part of the CST were excluded. We performed exclusion with *tckedit*. We, then, created mask images of the FA map by selecting all voxels passed by CST streamlines with *tckmap* and extracted the FA values within these CST volumes with *mrdump* [17].

We defined contact of the segmented CST volume with the tumor as infiltration, while a reduced volume of the CST compared to the contralateral hemisphere was defined as compression. Further, an asymmetric arrangement of both CSTs due to mass effect was defined as dislocation. If these features were absent, we considered the CST to be unaffected.

### 2.5. Statistics

All statistical analyses were performed in GraphPad Prism (version 8.4.0, San Diego, CA, USA). The normal distribution was tested after D′Agostino–Pearson. We analyzed the statistical differences with the Mann–Whitney U-test. Results with *p* < 0.05 were considered statistically significant. FA values above 1 or below 0 were excluded from the analysis, because they have to be considered false calculations due to noise. Values are given as the mean with the standard error of the mean (SEM). The fifth percentile was calculated to propose a future FA cutoff value.

### 2.6. Testing Different FA Cutoffs a Posteriori

To exemplify the impact of different FA cutoffs for the tractography results, we tested three different cutoff values in one CST with BrainLab iPlan Cranial 3.0. First was the lowest FA cutoff in the literature of 0.1 [6]. Second was the standard FA cutoff in BrainLab iPlan Cranial of 0.3. Then, we performed tractography with our proposed cutoff value. ROIs were placed as described above, and the minimum streamline length was set to 30 mm as described above. For visualization, volumes were rendered from the streamline results.

## 3. Results

### 3.1. Patients

Between March 2020 and January 2021, 14 patients were included (six male and eight female, 50.1 ± 4.0 years old). The most common diagnosis was glioblastoma of the left hemisphere. The demographic data are shown in Table 1.

### 3.2. FA Distribution in the Corticospinal Tracts

**Figure 1:**
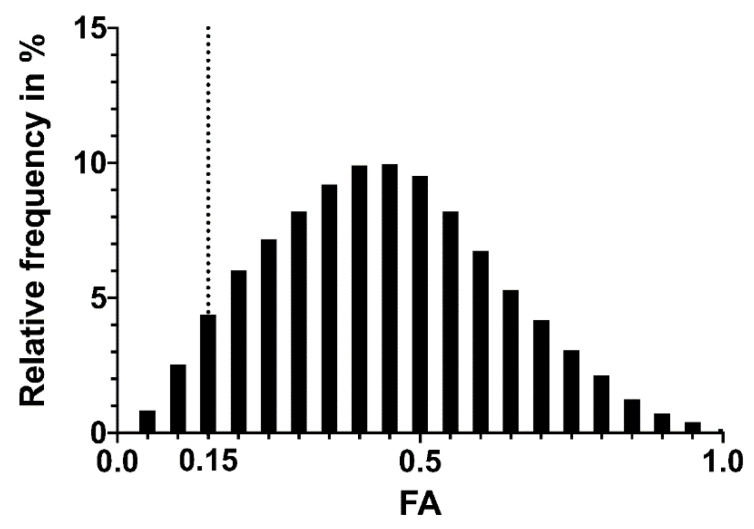
The frequency distribution of all fractional anisotropy (FA) values in our cohort (left, right, preop, and postop). Dotted line: 0.15. *n* = 293,921. The mean FA is 0.4406 ± 0.0003, the fifth percentile is 0.1454. The FA values are not normally distributed.

**Figure 2:**
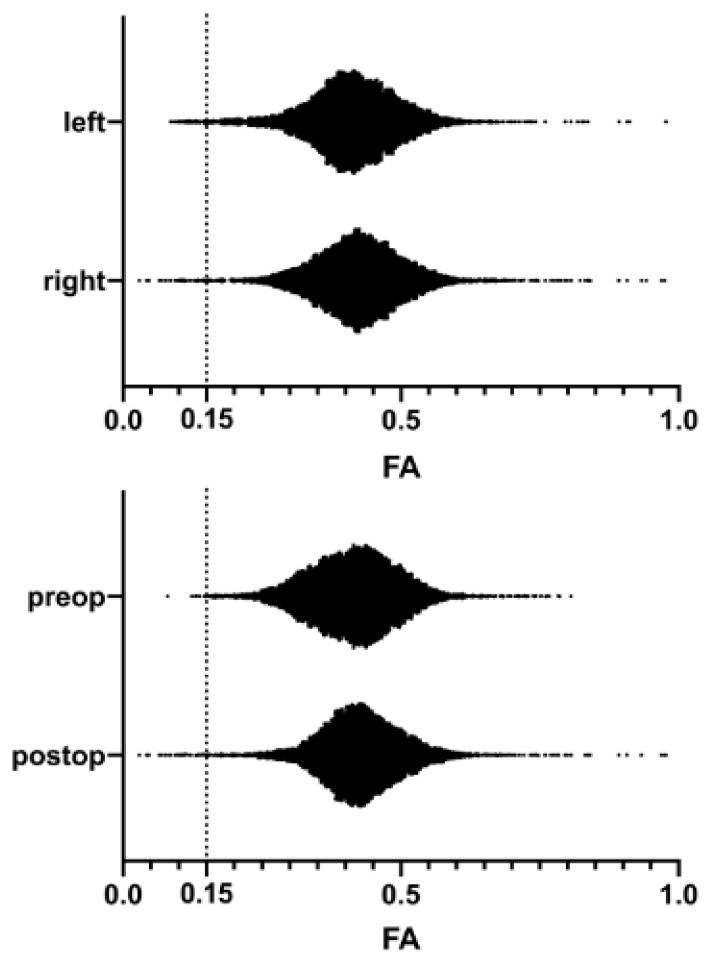
The pooled FA values in the left and right hemisphere (above) and preoperative and postoperative MRI (below). Dotted line: 0.15. There was a statistically significant difference between the CSTs of the left and right hemisphere (median 0.4156 versus 0.4263, *p* < 0.0001), as well as between the preop and postop FA (median 0.4198 versus 0.4266, *p* < 0.0001) with the postop FA values being especially less frequent below their mean.

Tractography resulted in 313,765 ± 24,016 streamlines per CST. The FA frequency distribution through all patients is shown in Figure 1. The values were not normally distributed (*p* < 0.0001). Altogether, 293,921 FA values are shown with a mean of 0.4406 ± 0.0003. The 95th percentile was at 0.7689, and the fifth percentile was at 0.1454. There was a statistically significant difference between the FA in the CST in the left and the right hemispheres (left 0.4379 ± 0.0005, *n* = 140,249; and right 0.4421 ± 0.0005, *n* = 149,807; *p* < 0.0001; Figure 2).

### 3.3. Postoperative Changes in the FA Distribution

Interestingly, we found a significant difference between the FA in all CSTs before and after surgery, with the postoperative FA values being more condensed around their mean (*p* < 0.0001, Figure 2). Preoperatively, the FA was 0.4390 ± 0.0005 (*n* = 145,130) with the fifth percentile at 0.1407. Postoperatively, the FA reached 0.4411 ± 0.0005 (*n* = 144,926) with the fifth percentile at 0.1490. The median increased from 0.4170 to 0.4262 after surgery.

### 3.4. Individual Distributions of FA Values

**Figure 3:**
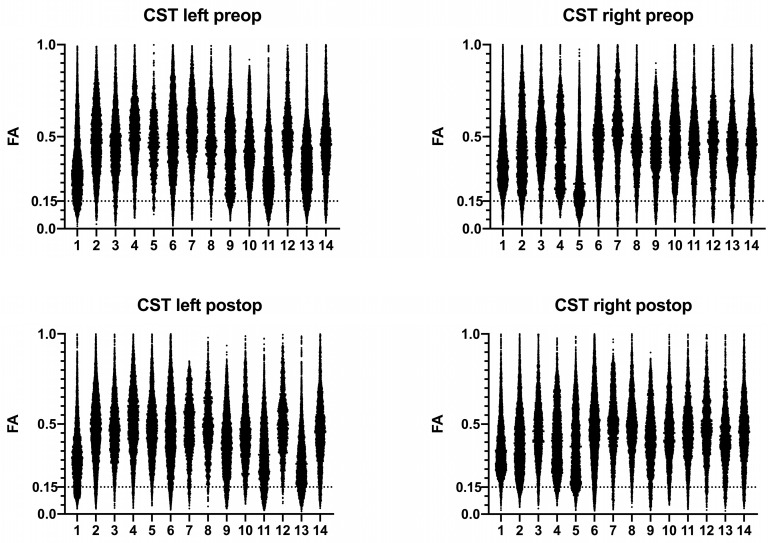
The individual distribution of the FA values in all patients before and after surgery. Dotted line: 0.15. The fifth percentile of every CST is given in Table 2. Note that especially infiltrated or compressed CSTs present an altered FA distribution toward the lower values. In infiltrated or compressed CSTs, the fifth percentile of FA values was 0.1407 ± 0.0109. In all other CSTs, the fifth percentile was 0.1763 ± 0.0040 (*p* = 0.0036).

The individual FA distributions for each patient and hemisphere before and after surgery are shown in Figure 3. The fifth percentile of the FA values in each reconstructed CST is given in Table 2. There was no statistically significant difference when comparing the fifth percentiles preoperative versus postoperative (0.1647 ± 0.0063 versus 0.1685 ± 0.0068, *p* = 0.5970), as well as when comparing the left versus right (0.1683 ± 0.0079 versus 0.1649 ± 0.0049, *p* = 0.5099).

### 3.5. Infiltrated or Compressed CSTs

For the analysis of CSTs compromised by the intracerebral tumor, we compared infiltrated or compressed CSTs against all others, which were (a) in the contralateral hemisphere, (b) only dislocated, or even (c) unaffected. In infiltrated or compressed CSTs, the FA was 0.3636 ± 0.0009, while the contralateral, dislocated, or unaffected CSTs showed an FA of 0.4442 ± 0.0007 (*p* < 0.0001). The mean of the fifth percentiles was 0.1407 ± 0.0109 in infiltrated or compressed CSTs and 0.1763 ± 0.0040 in all others (*p* = 0.0036).

### 3.6. Tractography Results at Different FA Cutoffs

**Figure 4:**
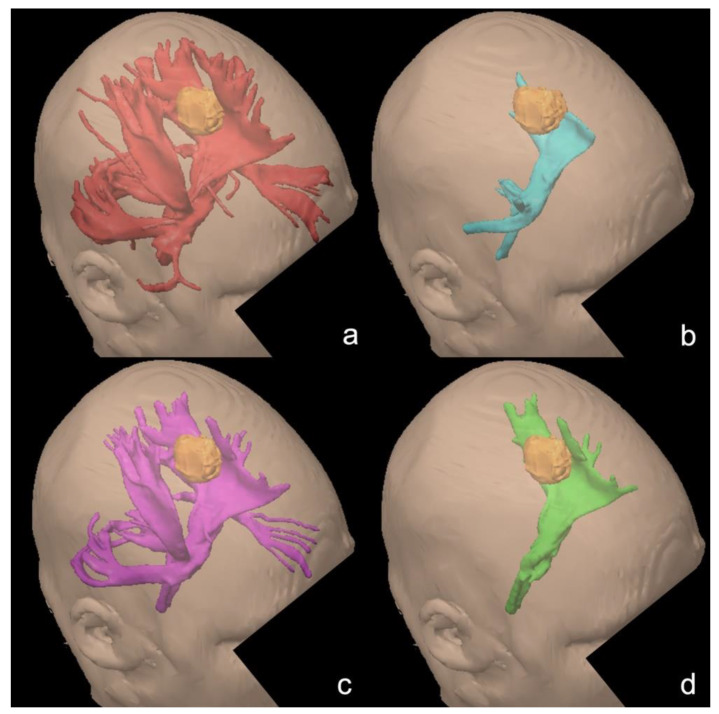
Illustrative case for tractography (iPlan Cranial 3.0, Brainlab AG, Munich, Germany) of an infiltrated CST in patient 11. For visualization, the streamlines have been rendered into volumes. Orange: tumor. (**a**): Tractography with the FA cutoff set to 0.1 (red). Many collaterals are included. (**b**): Tractography with the FA cutoff set to 0.3 (blue). In proximity to the cortex, the tractogram is cut off early. (**c**): Tractography with the FA cutoff set to 0.15 (purple). Collaterals to the cerebellum, the contralateral hemisphere, and the inferior fronto-occipital fascicle are included. (**d**): After exclusion of collaterals from the tractogram (4c, purple), an anatomically realistic tractogram remains (green). Infiltration of the CST is evident in all tractograms.

To exemplify the impact of different FA cutoffs on the tractography results, a tractogram from BrainLab iPlan Cranial 3.0 of the left CST in patient 11 is shown with different cutoff values (Figure 4). For visualization, the volumes have been rendered from the streamline results. While the lowest cutoff (0.1) delivers a tractogram with many anatomically unrealistic streamlines (Figure 4a), the standard cutoff (0.3) is missing a significant amount of anatomically realistic streamlines (Figure 4b). The cutoff value of 0.15, which reflects the fifth percentile of our data (Figure 4c), provides a robust result with some streamlines that can be interpreted as anatomically realistic collaterals. Figure 4d shows the final tractogram of the CST after the removal of these collaterals.

## 4. Discussion

In clinical practice, setting the FA cutoff value for tractography in planning neurosurgical procedures is one of the key technical factors. Consequently, a reliable cutoff value has to be determined that does not exclude plausible anatomical streamlines and yet preserves a reasonable threshold against technical noise. While good workflows exist that solve this task sufficiently, a reliable starting point could shorten these workflows. Hence, in this study, we analyzed the frequency distribution of the fractional anisotropy values in the corticospinal tracts of patients undergoing neurosurgery for intracerebral tumors. This patient collective is especially critical as mass effects or infiltration will reduce the directedness of diffusion and, thus, have a negative impact on tractography [15]. Our cohort is comparable to other studies on neurosurgical patients [20].

The analysis of the frequency distribution offers insight into the technical basis of the tractography workflow. It is crucial to keep in mind that a too high FA cutoff value might possibly exclude real anatomical parts of a fiber bundle. On the other hand, setting the cutoff value too low will result in too many false positive streamlines to interpret the actual anatomy of the patient. While there is a consciousness in the existing literature that the optimal FA cutoff value is unclear, our statistical approach may shorten the process of trial and error.

When searching for a reasonable FA cutoff value, we want to consider all FA values that appear within the anatomical volume of the respective fiber bundle. Therefore, we defined this volume with our tractography workflow. As almost all possible FA values appear in the resulting volume, every tractography algorithm requires an FA cutoff value. As streamline tractography stops below this value, it should be as low as possible. However, as many FA values from the calculated tractogram volume as possible should be above the FA cutoff value. Hence, we chose to search for the FA value that would leave 95% of all FA values from our tractogram volume above the cutoff value and calculated the fifth percentile of FA values for each CST volume. In our eyes, this is a reasonable way to find the threshold between the real anatomical FA values and noise.

The fifth percentile of the FA values in all groups is very close to 0.15, which we propose as the standard starting point FA cutoff in neurosurgical patients. However, we also found differences within our cohort. Postoperatively, the FA distribution in the CST was significantly more condensed around the mean, with the median increasing from 0.4193 to 0.4257 after surgery. We attribute this change to the effect of decompression, which relieves edema and restores homeostasis in the affected fibers [21].

We also found a significant difference between the CSTs of the left and the right hemisphere, with higher FA values on the right side. This effect most likely stems from the fact that only 3 of 14 patients suffered from tumors in the right hemisphere. Patients with tumors of the left and dominant hemisphere may have been included more frequently into our cohort because of a clinical interest in functional imaging.

An important question is whether the FA threshold of 0.15 applies to all patients in our cohort. Therefore the fifth percentile in every CST before and after surgery was calculated. The comparison between left and right and comparison between preoperative and postoperative MRI revealed no statistically significant differences. However, when comparing only the compressed or infiltrated CSTs against all others, the mean of the fifth percentiles was significantly lower at 0.1407. Although this is acceptably close to a threshold of 0.15, even a cutoff of 0.1 might be necessary for some patients, as Table 2 indicates. Nevertheless, Figure 4d shows an illustrative result based on our proposed workflow.

### Limitations

This is an observational study with no randomization for patient inclusion. Therefore, eloquent lesions are overrepresented. Further, few FA values below 0 or above 1 had to be excluded, which is proof of some noise in the image acquisition as the FA can only be within these limits. Although our data do not suggest it, this could impact the reproducibility of the MRI sequence.

Like every tractography workflow, our study relies on anatomical verification by experienced clinicians. In particular, the exclusion of collateral fibers or noise could be a possible source for false-negative results. We also did not use the neurophysiological localization of primary functional areas by transcranial magnetic stimulation or direct electrical stimulation. Therefore, the actual function of the displayed and analyzed fiber bundles remains implicit.

The tractograms in Figure 4 were derived using BrainLab iPlan Cranial 3.0, which uses the deterministic FACT algorithm [22]. In contrast, we used a probabilistic tractography algorithm to define the CST volumes in the first place. In our opinion, this is justified, because the analyzed FA data itself is independent of the tractography algorithm. Reproduction of our workflow across platforms is possible, as our results show. However, we only applied a single diffusion MRI protocol. Reproducibility with other MRI sequences must be shown in further studies [2].

Further, the sample size of patients in our study was relatively small. Although we believe that Table 2 shows the sample is representative for neurosurgical patients, this might lessen the importance of our results. However, the raw data in Figure 3 as well as the applied statistics of altogether 293,921 FA values demonstrated statistically significant results.

## 5. Conclusions

Our data show that 0.15 was a reasonable FA cutoff value in tractography for neurosurgical patients with intracerebral lesions, which can be used as a starting point for other tractography workflows. For infiltrated fiber bundles, a cutoff of 0.1 should be taken into consideration. Tractography remains a patient-tailored technique in neurosurgical planning.

## Figures and Tables

**Table 1:** Demographic data.

Patient	Age (Years)	Gender	Diagnosis	Side	Lobe	CST Affection
*1*	54	M	Glioblastoma WHO grade IV	Left	Parietal	Compressed
*2*	33	M	Glioblastoma WHO grade IV	Right	Parietal	Dislocated
*3*	42	M	Oligodendroglioma WHO grade III	Left	Frontal	Unaffected
*4*	65	F	Metastasis	Right	Occipital	Compressed
*5*	36	F	Glioblastoma WHO grade IV	Right	Parietal	Compressed
*6*	69	M	Metastasis	Left	Frontal	Unaffected
*7*	40	F	Glioblastoma WHO grade IV	Left	Frontal	Dislocated
*8*	63	F	Meningioma WHO grade III	Left	Parietal	Compressed
*9*	70	M	Glioblastoma WHO grade IV	Left	Temporal	Compressed
*10*	67	F	Glioblastoma WHO grade IV	Left	Temporal	Compressed
*11*	32	F	Astrocytoma WHO grade III	Left	Frontal	Infiltrated
*12*	30	F	Astrocytoma WHO grade I	Left	Temporal	Unaffected
*13*	45	F	Glioblastoma WHO grade IV	Left	Frontal	Infiltrated
*14*	55	M	Glioblastoma WHO grade IV	Left	Temporal	Dislocated

**Table 2:** The fifth percentile of the FA values in each reconstructed CST. The side of the affected hemisphere and the CST affection are given for comparison. Note that infiltrated or compressed CSTs had a significantly lower fifth percentile of FA values (0.1407 ± 0.0109 versus 0.1763 ± 0.0040, *p* = 0.0036).

Patient	Side	CST Affection	Preop Left	Postop Left	Preop Right	Postop Right
*1*	Left	Compressed	0.1153	0.1076	0.1653	0.1701
*2*	Right	Dislocated	0.1897	0.1744	0.1567	0.1517
*3*	Left	Unaffected	0.1559	0.2098	0.1714	0.1923
*4*	Right	Compressed	0.2148	0.21.04	0.1697	0.1577
*5*	Right	Compressed	0.2068	0.2094	0.0812	0.1314
*6*	Left	Unaffected	0.1783	0.1542	0.1641	0.1476
*7*	Left	Dislocated	0.2296	0.1798	0.1759	0.1945
*8*	Left	Compressed	0.2128	0.2113	0.1811	0.2162
*9*	Left	Compressed	0.1514	0.1278	0.1960	0.1422
*10*	Left	Compressed	0.1674	0.1704	0.1471	0.1761
*11*	Left	Infiltrated	0.1022	0.0930	0.1871	0.1891
*12*	Left	Unaffected	0.1547	0.2200	0.1458	0.1916
*13*	Left	Infiltrated	0.3663	0.0944	0.1665	0.1453
*14*	Left	Dislocated	0.1595	0.1886	0.1430	0.1601

## Data Availability

Data of this work is available from the corresponding author upon reasonable request.
